# A new type of pictorial database: The Bicolor Affective Silhouettes and Shapes (BASS)

**DOI:** 10.3758/s13428-021-01569-7

**Published:** 2021-05-07

**Authors:** Claudia Kawai, Gáspár Lukács, Ulrich Ansorge

**Affiliations:** 1 Department of Cognition, Emotion, and Methods in Psychology, University of Vienna, Vienna, Austria; 2 Vienna Cognitive Science Hub, University of Vienna, Vienna, Austria; 3 Research Platform Mediatized Lifeworlds, University of Vienna, Vienna, Austria

**Keywords:** Affective rating, Silhouette, Emotion, Database, Valence, Arousal, Online research

## Abstract

We introduce the Bicolor Affective Silhouettes and Shapes (BASS): a set of 583 normed black-and-white silhouette images that is freely available via https://osf.io/anej6/. Valence and arousal ratings were obtained for each image from US residents as a Western population (*n* = 777) and Chinese residents as an Asian population (*n* = 869). Importantly, the ratings demonstrate that, notwithstanding their visual simplicity, the images represent a wide range of affective content (from very negative to very positive, and from very calm to very intense). In addition, speaking to their cultural neutrality, the valence ratings correlated very highly between US and Chinese ratings. Arousal ratings were less consistent between the two samples, with larger discrepancies in the older age groups inviting further investigation. Due to their simplistic and abstract nature, our silhouette images may be useful for intercultural studies, color and shape perception research, and online stimulus presentation in particular. We demonstrate the versatility of the BASS by an example online experiment.

Images are an excellent medium to convey information, simple or complex. They serve as stimuli in a wide range of research areas, such as emotion, attention, or aesthetics research, among others (e.g., Bradley & Lang, 2007; Huston et al., 2015; Lindsay, 2020). In the present article, we introduce an open access database of normed Bicolor Affective Silhouettes and Shapes (BASS).^1^ Each of the BASS images consists of 300 × 300 black and white pixels, providing a computationally economical and visually uniform layout.^2^

New freely available normed images enrich research by providing greater diversity of choices for research, including potential replications of findings with different kinds of images to ensure generalizability. Additionally, participants may get habituated to the images in existing databases, meaning they may process them differently on repeated exposures (Baker et al., 2010; Foa & Kozak, 1986; Ramaswami, 2014). The present BASS database may be used, for instance, for conceptual replications and novel research in such diverse areas as space-valence congruence effects (e.g., Meier & Robinson, 2004), priming of evaluations (e.g., Fazio, 2001), affective priming (e.g., Hermans et al., 2001), or emotional facilitation (e.g., Schupp et al., 2003).

More importantly, the BASS also has very specific advantages compared to other available stimulus sets: Silhouette images allow for the study of meaning-related processing without many of the confounds that are present in words and pictures. Whereas words carry inherent differences, such as their length and their phonological transparency (or orthographic depth, see Aro & Wimmer, 2003; Frost et al., 1987; Schmalz et al., 2016) for which one would have to control, these confounding differences are absent in silhouettes. Likewise, whereas pictures differ from one another in terms of color heterogeneity, depth cues, spatial perspective, or feature complexity, silhouettes are more uniform and less complex, and can therefore be more easily equated for these factors, without corrupting their meaning entirely.

The main purpose of the present research article is to demonstrate that the BASS images evoke a very wide range of affective representations on the side of the participants—despite the images’ relative simplicity. We demonstrate this primarily by collecting valence and arousal ratings in a large and nationally representative US (Western) sample. We provide further evidence from analogous ratings from an extensive (Eastern) sample from Mainland China and from demonstrating characteristic valence-color congruence effects in an example response time experiment using BASS images (in an Austrian sample).

This methodological approach validates several important characteristics of the BASS images, relevant to but often not investigated in image sets used in psychological research. First, typically, normative ratings of affective picture databases like the Geneva Affective Picture Database (GAPED), the Open Affective Standardized Image Set (OASIS), the International Affective Picture System (IAPS), or the Nencki Affective Picture System (NAPS) (Dan-Glauser & Scherer, 2011; Kurdi et al., 2017; Lang et al., 1997; Marchewka et al., 2014, respectively) are largely based on mono-cultural samples, with ratings predominantly collected in Western populations only. Moreover, within Western populations, samples were often restricted to student participants.^3^ Yet, ideally, behavioral image or stimulus sets should be applicable in different cultures in order to allow investigation of universal psychological phenomena and topics. The IAPS, for example, has been generally found to be valid among Western cultures (Deák et al., 2010; Grühn & Scheibe, 2008; Verschuere et al., 2001, to name a few), but less so among Asian cultures (Gong & Wang, 2016; Huang et al., 2015). Therefore, from the start, for the current BASS images, we intended to collect normative rating data from at least two large, representative samples of different cultural origin: one Western (US) and one Eastern (Mainland China) sample. Silhouette images theoretically carry potential for more culturally neutral affective representations than photographic images, due to the latter’s culture-specific details (such as depictions of regionally different vegetation, landscapes, food, people, architecture, traffic etc.), which are often subtle and difficult to control for. We tested this conceptual notion by directly comparing the consistency of the ratings from our Western and Asian samples.

Second, the bicolor silhouettes tackle one particular virulent problem posed by online studies: accurate color rendition of heterogeneous colors on different physical displays. By implication of their high realism, color photos of natural scenes carry substantial color heterogeneity and, in fact, their realism relies in part on this color heterogeneity (cf. Hansen & Gegenfurtner, 2009). It has indeed been shown that differences in the display of photographs, such as varying brightness or resolution, can substantially affect the evaluation of emotional content (e.g., Lakens et al., 2013; Mould et al., 2012). However, the huge variety of physical devices used to display images (e.g., smartphones, tablets, and laptops by different manufacturers) plus an increased variance in idiosyncratic monitor settings and lighting conditions on the side of the viewer compromise accurate color rendition, especially in online studies (Anwyl-Irvine et al., 2020)—a problem which increases with the complexity of the image’s color palette. In contrast, the black-and-white silhouettes of the BASS database were already created and selected (from existing silhouettes) under the premise of having to convey their affective meanings largely by their contours alone and less by their exact color rendition. In this way, the BASS images reduce the heterogeneity of the necessary color palette for their rendition and the problems associated with this color heterogeneity. We demonstrated this by collecting participants’ affective ratings using the BASS images in inverse colors (pilot study).

However, this is not to say that colors could not be manipulated and used to study their effects (e.g., on affective ratings) in studies with BASS images. For photographs, manipulations of color rendition can easily corrupt image meaning altogether (cf. Oliva & Schyns, 2000; Mould et al., 2012). Such manipulation requires complicated technical procedures (e.g., Orzan et al., 2007), and, even more fundamental, one can hardly be sure what manipulation of colors in a photography affects emotional image content in what manner: Setting up a proper balance of hue, saturation, and brightness is difficult enough when calibrating the display of a single color (Wilms & Oberfeld, 2018), let alone for a photograph with thousands of interacting colors. In contrast, the BASS images were created with the intention of keeping an unambiguous meaning by way of their contours, even if presented in varying colors. Thus, BASS images offer an extremely easy way to manipulate and study or control color: They can be converted effortlessly to any other two colors^4^—and, as long as the two colors are discernible from each other, the content remains unambiguous. Below, we also demonstrate how such color manipulations of the BASS images could be used in research by investigating known color-valence congruence effects using variously colored BASS images for an example experiment.

Third, large photographic images are generally not optimal for fast decision tasks based on their diverse visual characteristics and complex semantics: Various depicted details and their many subtle visual properties might affect each participant very differently, influencing processing time and potentially confounding results (especially where small effects are expected and processing-time differences in fractions of seconds can affect outcomes). In contrast, the contents of the less ambiguous (few or single) objects in each BASS image is easy to grasp quickly and better suited for a task involving fast decisions, unaffected by visual or semantic noise. In addition, their simplistic and abstract nature makes BASS silhouettes more resistant than photographs to incidental but potentially meaning-corrupting influences such as display size or viewing distance (Anwyl-Irvine et al., 2020).^5^ Big file sizes of photographic images used in experiments typically pose technical problems too, especially in connection with online research, which is becoming more and more important in psychological science. For one, common browsers are not optimized for precisely timing image presentation, and physical display times can be strongly affected by loading large size images (e.g., Garaizar & Reips, 2019). For another, some participants might be reluctant (or even unable, where infrastructure is weak) to download many large files. It is not unusual for an experiment to require several hundreds of different stimuli (especially when each stimulus is presented repeatedly in different forms, e.g., varying hue, etc.; e.g., Huang et al., 2015; Kawai et al., 2020; Oliva & Schyns, 2000). Calculating with the average file size of IAPS images (262 KB; cf. averages of 92 KB for OASIS and 789 KB for GAPED), just 100 images take 26 MB—already a notable hurdle for potential participants. Participant dropout due to inadequate internet speed could even introduce confounds via selective attrition (Zhou & Fishbach, 2016). With an extremely economical average file size of 2.2 KB (max. 6.0 KB), the BASS images minimize the issues of display timing and download. Our follow-up experiment on color-valence congruence effects also serves to demonstrate these usability advantages in fast decision tasks online.

Fourth, silhouettes, contours, and shapes themselves constitute a field of scientific interest (e.g., Bar, 2007; Biederman, 1987; Marr, 1982), and the affective qualities associated with geometric contour properties may be of interest to future researchers (e.g., Leder et al., 2011), although they have not been a major focus of research on contours so far. This could be particularly interesting when comparing human object recognition with artificial object recognition (e.g., Rajalingham et al., 2018): While objects as simple as silhouettes may be processed by machines similarly to humans, affective responses are (currently) only evoked in humans.

## Creating the BASS

Before carrying out the large-scale ratings for the BASS, we assessed several issues in a pilot study (*n* = 180, students of the University of Vienna). Detailed information on the pilot study can be found in the online supplementary material available on https://osf.io/anej6/. Firstly, researchers suspected, but never tested, possible contamination of affective ratings when participants had to rate valence and arousal in one experiment (Kurdi et al., 2017), since prior studies showed that question order in surveys can influence judgements (Lau et al., 1990; Wilcox & Wlezien, 1993). We put this assumption to the test and split participants into two groups: one rating only valence (or only arousal) and another rating images on both valence and arousal. Results indeed confirmed contamination in affective ratings when two affective ratings were required in the same experiment. Thus, we decided to use only one rating measure (either valence or arousal) per participant in the main study. In the pilot study, we simultaneously tested for the relative-color independence of the silhouette ratings by studying possible effects of color-inversion of silhouettes (black-on-white vs. white-on-black) on ratings. High positive correlations between the (original) black-on-white images and the inverted white-on-black images suggested that there is no apparent preferred color mode for ratings of our silhouettes (clarity ratings: *r*[618] = .866, 95% CI [.845, .885]; valence: *r*[618] = .918, 95% CI [.905, .930]; arousal: *r*[618] = .719, 95% CI [.678, .755]). The ratings from the pilot study, given by Austrian psychology students, also helped us to decide on a reasonable exclusion threshold for intrarater correlation, which we subsequently used as an individual reliability measure for data quality control in the main study. Since intrarater correlation turned out to be lower in the arousal rating than in the valence rating condition, we determined different exclusion thresholds depending on the rating condition (see Data Exclusion).

Clarity ratings provided by the first as well as a second online prestudy (*n* = 50, Chinese participants) helped to identify unclear or ambiguous images in the original set of 620. After excluding those with the lowest clarity ratings, the subset that was used in the large-scale ratings in the US and China comprised a total of 583 silhouettes.

## Method

### Participants

For the US rating, a total of 806 participants was recruited via Prolific (www.prolific.co). The study was divided into two parts: one sub-study comprised the valence rating (*n* = 402), the other the arousal rating (*n* = 404) task. The sample was collected as a “representative sample for the United States of America” (sex, age, ethnicity, according to Simplified US Census; excluding people under the age of 18) and participants received £2.17 upon study completion.

The experiment had to be completed at a desktop computer. Participants could read all relevant information on the welcome page and consented to participate by clicking the consent button at the bottom.

After exclusion (see Data Exclusion), valid data from 777 participants remained (arousal rating: 386; valence rating: 391), out of which 402 participants were female, 375 were male. Ages ranged from 18 to 78 years, with a mean age of 45.19 years (*SD* = 16.09).^6^ Completion took an average of 17 min (*SD* = 4 min, median = 15 min).

For the Chinese sample, the recruitment (and compensation) of online participants in China was managed by Dynata. In the supplementary materials, we provide more information on Dynata’s incentive and compensation system. Chinese participants were asked to provide demographic data after consenting to the study. We only allowed participation for those who indicated (1) their age being between 18 and 95 years, (2) Mainland China as their country of residence, and (3) any gender information. A total of 1341 registered participants submitted their rating data, of which 472 had to be excluded due to poor data quality (for more details see Data Exclusion). We aimed for a representative sample (sex; age, excluding people under the age of 18 years) for the People’s Republic of China, but since our study required PC and Internet access, relatively younger age groups are over-represented.

After exclusion, valid data from 869 participants remained (arousal rating: 455; valence rating: 414), out of which 419 participants were female and 450 were male. Ages ranged from 18 to 81 years, with a mean age of 34.5 years (*SD* = 9.4). Completion took on average 25 min (*SD* = 116 min, median = 14 min).^7^

An overview of the demographic data from all included participants (US and China) is provided in Table 1 (Appendix A).

### Materials

The majority of the silhouettes were acquired using Google Images (images.google.com) under the creative commons license, by searching either directly for silhouettes or for photographic pictures that we edited into black-and-white silhouettes using GNU Image Manipulation Program (The GIMP Development Team, 2019) and R (R Core Team, 2020).

We restricted our search to images labeled “available for reuse with modification.” Most silhouettes are from pixabay.com (*n* = 444), cleanpng.com (*n* = 33), and svgsilh.com (*n* = 29). Eighteen additional silhouettes were created for this project by a professional illustrator. We collected a wide range of images, depicting humans, animals, objects, and scenes, which we indicated by a category column in the database for easier stimulus selection. Special focus was put on collecting images of a wide range of valence (positive, negative, and neutral) as well as arousal (low, medium, and high) levels. Every image was scaled and/or cropped to a size of 300 × 300 pixels. All images were checked (or converted) to contain only fully black (RGB: 0, 0, 0) and fully white (RGB: 255, 255, 255) pixels.

### Procedure

#### Online Rating Study – US

After giving their informed consent, participants saw the instruction page with an explanation of the terms valence and/or arousal (see Figs. 11 and 12 in Appendix A), depending on the participants’ rating condition, and three example images (selected based on the pilot studies as average in both arousal and valence; none of these images were used in the subsequent task for the given participant). The experiment started as soon as the “Start” button on the bottom of the instruction page was pressed. Each black-and-white silhouette stimulus (300 × 300 px) was presented on a grey background (RGB: 128, 128, 128) for 2 s. When this time had elapsed, the image disappeared and was replaced with the rating scale, a line ranging from “very low” on the left to “very high” on the right, with nine equally spaced tick marks (see Fig. 13 in Appendix A). Participants could enter their ratings by clicking on the scale(s) and submit their rating by pressing a button that appeared below, or they could skip the rating. After confirming their choice, the next image appeared on the screen, and so on. A total of 145-146 silhouettes were presented to each participant. Additionally, we incorporated two attention checks. Hyperlinks to the original experimental websites are available via https://osf.io/anej6/.

#### Attention checks

After rating every stimulus in the list, a black-and-white image was shown after which the participant had to indicate what was depicted on it out of a list of answers (this was the same item for all participants, a clearly discernible black-and-white image of a car). In addition, we assessed participants’ attention via intrarater reliability. Following the first attention check, the participant was presented with five of their most highly and five of their most lowly rated images (in randomized order). We calculated Spearman’s correlation coefficient between the first and second ratings for these ten items.

#### Online Rating Study – China

The procedure of the experiment was identical to that used for the US sample, with the exception that Chinese participants were asked to provide demographic data (gender, age, country of residence) after giving consent to the study.^8^ The experimental website including the informed consent and instruction pages was translated into Mandarin Chinese by a native speaker (a psychologist) and independently double-checked by another native speaker (a linguist).

### Data exclusion

Participants were excluded according to the following criteria: (1) the rate of skipped answers was above 25%; (2) no intrarater correlation could be calculated;^9^ (3) for arousal ratings, a failed Attention Check 1 and an intrarater correlation of less than .77 or otherwise a passed Attention Check 1 but an intrarater correlation of less than .67; (4) for valence ratings, a failed Attention Check 1 and an intrarater correlation of less than .80; or otherwise a passed Attention Check 1 but an intrarater correlation of less than .70.^10^ In addition, in the Chinese rating, data from participants was excluded if they did not provide a personal identification code (generated by Dynata).

Surprisingly, exclusion rates for the China data (472/1341 = 35.2%) were much larger than that for the US data (29/806 = 3.6%). This may have happened for various reasons, but a major difference is that Prolific (US data) specializes in collecting data for scientific purposes, while Dynata serves mainly as a provider of market research data.

## Results

Each image received between 91 and 119 ratings. For each country, we computed mean valence and mean arousal ratings per image.

### US rating

#### Reliability

For valence and arousal ratings, separately, we generated 1000 random split halves of our sample and calculated the Spearman–Brown reliability coefficient of the mean ratings. The mean of the correlation coefficients in the valence dimension was ${\overline{R}}_{val}$ = .986 (*SD* = 5.51 × 10^-5^, range: *R*_*min*_ = .985 and *R*_*max*_ = .986), and in the arousal dimension ${\overline{R}}_{aro}$ = .921 (*SD* = 3.50 × 10^-4^, range: *R*_*min*_ = .920 and *R*_*max*_ = .929), demonstrating extremely high reliability in both affective dimensions.

#### Relationship between valence and arousal

**Fig. 1:**
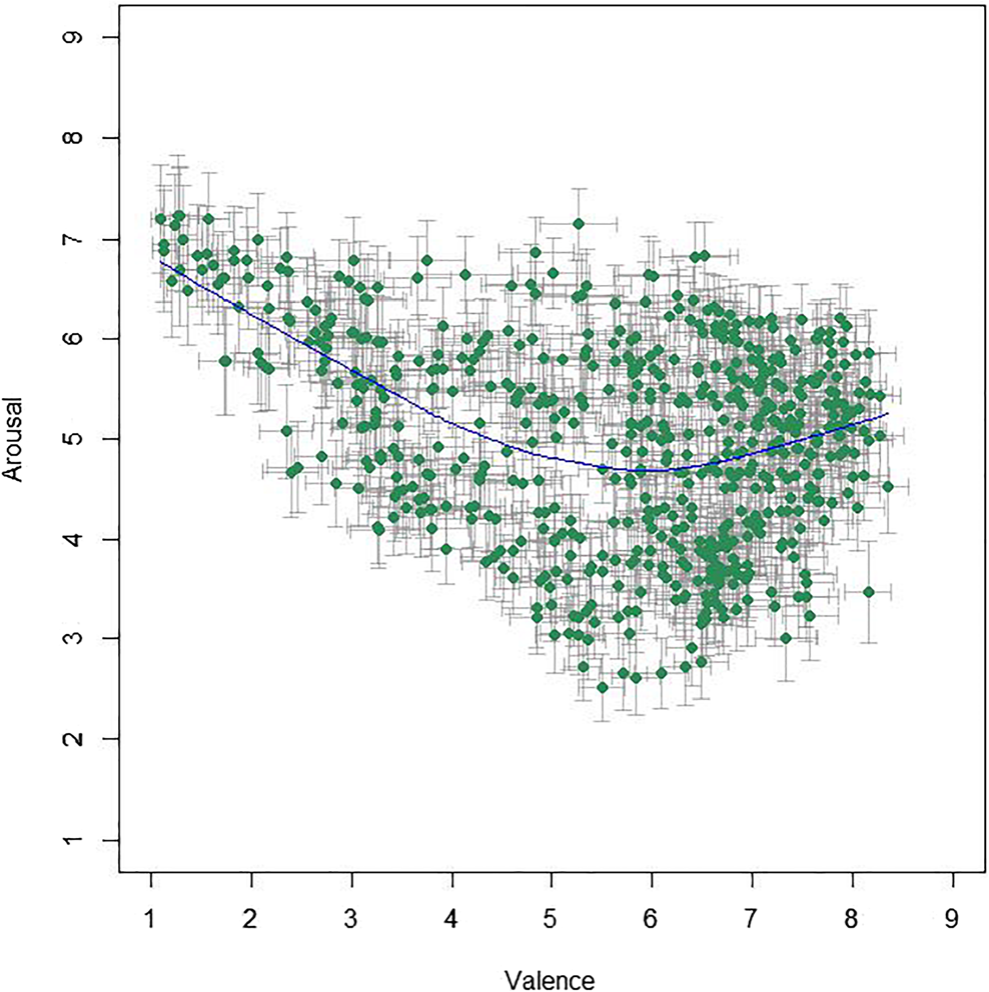
Mean ratings (95% CI error bars) per image for valence and arousal from US participants. To illustrate the tendency of the relation, the *blue line* depicts locally weighted regression (LOWESS)

Mean valence ratings from the US participants lie between 1.11 and 8.35 (*M* = 5.64, *SD* = 1.76); mean arousal ratings lie between 2.51 and 7.23 (*M* = 5.00, *SD* = 1.02). The correlation between valence and arousal ratings was significant, with *r*(581) = – .323, 95% CI [– .394, – .248], *p* < .001, *BF*_10_ = 5.76 × 10^12^. However, the Pearson correlation coefficient shows a relatively weak linear relationship and a look at the distributions of the US mean ratings, as shown in Fig. 1, suggests a U-shaped relationship between valence and arousal in the BASS images, where arousal dips for neutral images but then increases with rising valence.^11^

#### Effects of demographic variables

Prior research has identified differences in emotion processing based on factors like gender (Bradley et al., 2001; Proverbio et al., 2009; Sabatinelli et al., 2004; Wrase et al., 2003) and age (Bradley & Lang, 2007; Grühn & Scheibe, 2008; Pôrto et al., 2010). To investigate potential gender-related differences in our affective ratings, we calculated the individual means for valence and arousal ratings for male and female US participants.

The correlation between the mean ratings made by male and female participants was very high, for both valence, *r*(581) = .972, 95% CI [.967, .976], *p* < .001, *BF*_10_ > 10^200^, and arousal, *r*(581) = .877, 95% CI [.857, .895], *p* < .001, *BF*_10_ = 2.31 × 10^182^.

**Fig. 2:**
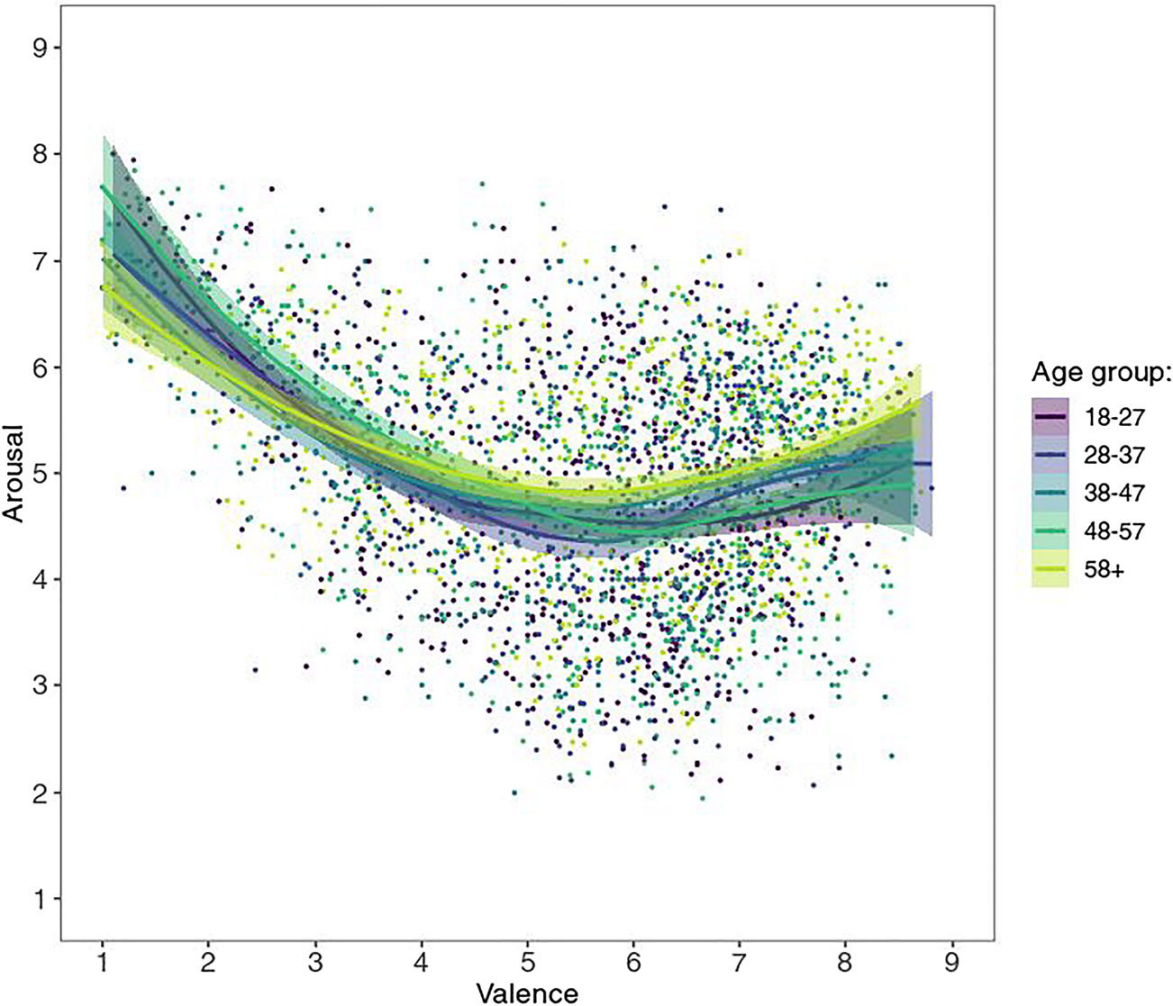
Mean valence by mean arousal ratings per picture by age group from US participants, with corresponding LOWESS lines

For age-wise comparison, we performed a median split for the demographic variable age on both of our US subsamples (valence, arousal) and correlated the respective mean ratings of the halves. For the valence rating group (median age = 46 years), correlation between the younger (< 46 years) and the older (≥ 46 years) age group was very high, with *r*(581) = .976, 95% CI [.972, .979], *p* < .001, *BF*_10_ > 10^200^. The same was true for the arousal rating group (median age = 44.5 years), with a correlation between the younger (< 45 years) and the older (≥ 45 years) age group of *r*(581) = .906, 95% CI [.891, .920], *p* < .001, *BF*_10_ > 10^200^. Finally, Fig. 2 shows that the valence-arousal pattern is very similar for all age groups (divided into five brackets following Prolific’s age stratification, see Table 1; see also Fig. 14 in Appendix A).

### Chinese rating

#### Reliability

The mean of the correlation coefficients in the valence dimension was ${\overline{R}}_{val}$ = .980 (*SD* = 7.64 × 10^-5^, range: *R*_*min*_ = .979 and *R*_*max*_ = .980), and in the arousal dimension ${\overline{R}}_{aro}$ = .921 (*SD* = 2.87 × 10^-4^, range: *R*_*min*_ = .917 and *R*_*max*_ = .923). Interrater reliability for both affective dimensions was again extremely high and almost identical to the US sample.

#### Relationship between valence and arousal

**Fig. 3:**
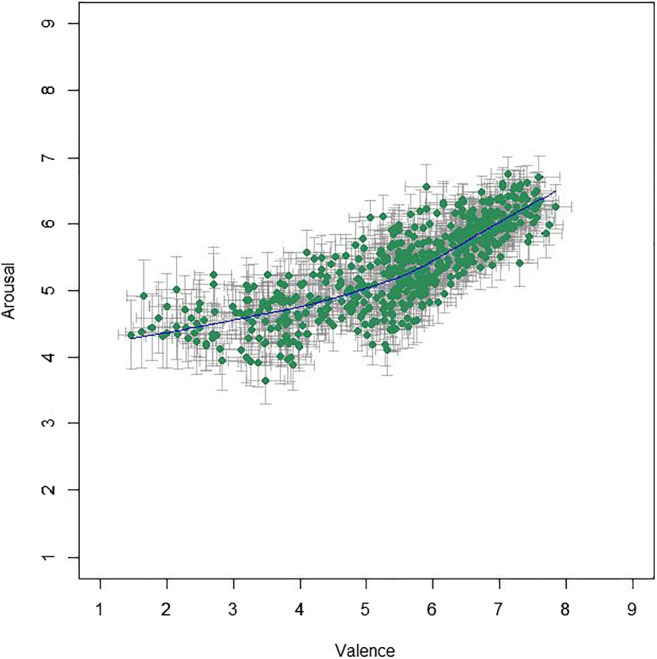
Mean ratings (95% CI error bars) per image for valence and arousal from Chinese participants with the LOWESS line in *blue*

Mean valence ratings from the Chinese participants lie between 1.47 and 7.84 (*M* = 5.46, *SD* = 1.37); mean arousal ratings lie between 3.65 and 6.75 (*M* = 5.31, *SD* = 0.64). Unexpectedly, and different from the results in the US sample, mean arousal and mean valence ratings showed a strong positive correlation, with *r*(581) = .805, 95% CI [.774, .832], *p* < .001, *BF*_10_ = 2.33 × 10^129^. The distributions of the Chinese mean valence and arousal ratings are shown in Fig. 3, which illustrates that negative images received relatively low arousal ratings while positive images received relatively high arousal ratings (as opposed to the US ratings, where arousal ratings were distributed fairly evenly between positive and negative images).

#### Effects of demographic variables

Similarly to the US sample, mean ratings between Chinese male and female participants were highly correlated for valence, *r*(581) = .979, 95% CI [.975, .982], *p* < .001, *BF*_10_ > 10^200^, and arousal, *r*(581) = .856, 95% CI [.832, .876], *p* < .001, *BF*_10_ = 4.11 × 10^163^.

For age-wise comparison, we again performed a median split for age on both Chinese subsamples (valence, arousal). For the valence rating group (median age = 32 years), correlation between the younger (< 32 years) and the older (≥ 32 years) age group was extremely high, with *r*(581) = .974, 95% CI [.970, .978], *p* < .001, *BF*_10_ > 10^200^. For the arousal rating group (median age = 33 years), the agreement between the younger (< 33 years) and the older (≥ 33 years) age group was still very high, although somewhat lower, *r*(581) = .785, 95% CI [.752, .814], *p* < .001, *BF*_10_ = 3.41 × 10^118^. Note that the median age was much lower in the Chinese compared to the US sample: The Chinese sample was split in the early 30s while the US sample was split in the mid-40s.

**Fig. 4:**
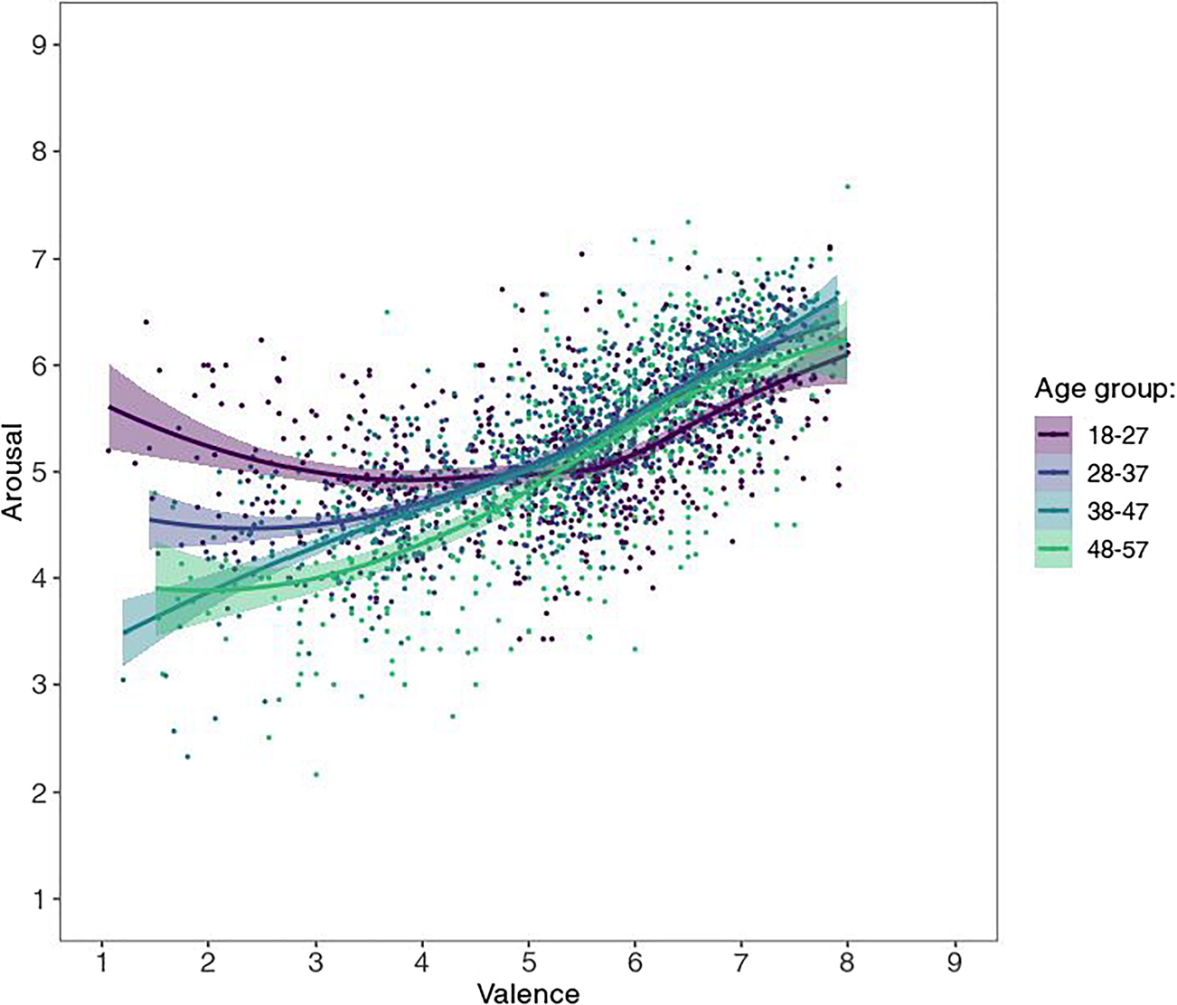
Mean valence by mean arousal ratings per picture by age group from Chinese participants, with corresponding LOWESS lines

As for the US sample, five age brackets were created—however, the ratings by Chinese age group “58+” are hardly reliable since this sample consists of a mere 14 participants—therefore they are omitted from the present figures. (They are nonetheless largely in line with the rest of the data; see supplementary figures at https://osf.io/anej6/). Most interestingly, the different age groups show somewhat different patterns (see Fig. 4), with ratings from younger age groups, especially the youngest (ages 18–27), tending to resemble more to the US sample and the U-shape often observed in Western samples.

### Comparison of US and Chinese ratings

**Fig. 5:**
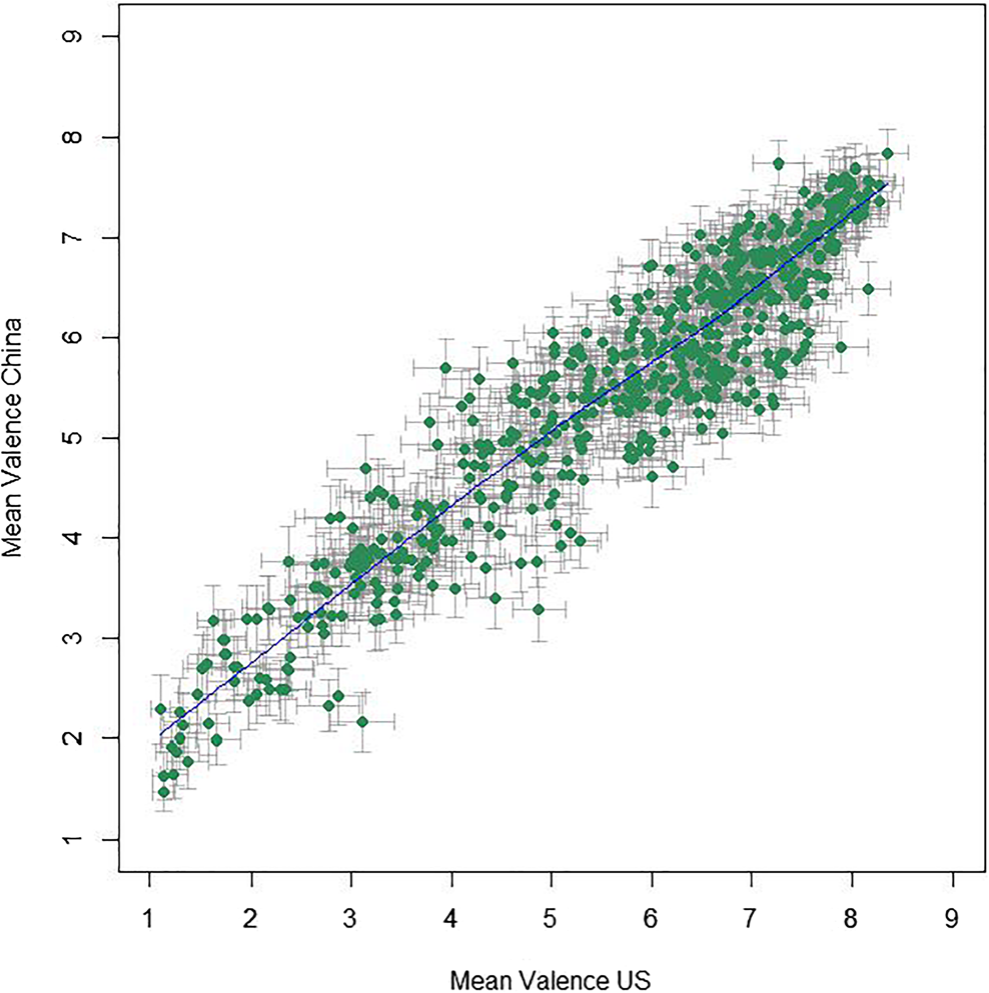
Relationship between US and Chinese mean valence ratings (95% CI error bars) per image, with the LOWESS line in *blue*

**Fig. 6:**
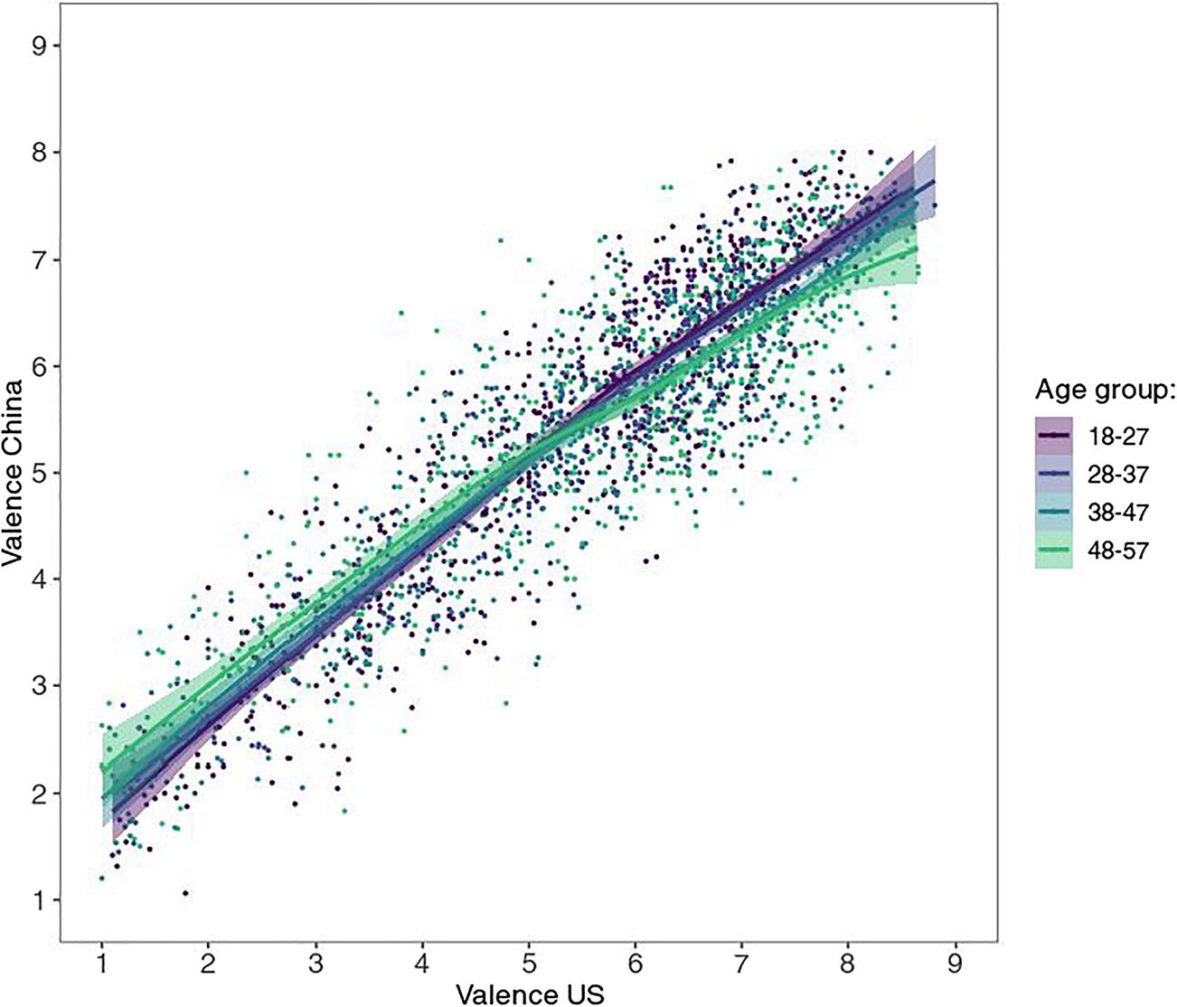
Relationship between US and Chinese mean valence ratings per image by age group, with corresponding LOWESS lines

Mean ratings in the valence category from the US sample showed extremely high positive correlation with those from the Chinese rating, *r*(581) = .936, 95% CI [.925, .946], *p* < .001, *BF*_10_ > 10^100^ (see Fig. 5). A paired *t* test showed that Americans rated images more positive than Chinese, with a statistically significant but practically negligible difference of 0.18, 95% CI [0.12, 0.23] (US: 5.64 ± 1.76, China: 5.46 ± 1.37), *t*(582) = 6.35, *p* < .001, *d* = 0.26, 95% CI [0.18, 0.35], *BF*_10_ = 1.21 × 10^7^. Both these findings are very similar for all age groups and both sexes (see Fig. 6; Table 2).

**Fig. 7:**
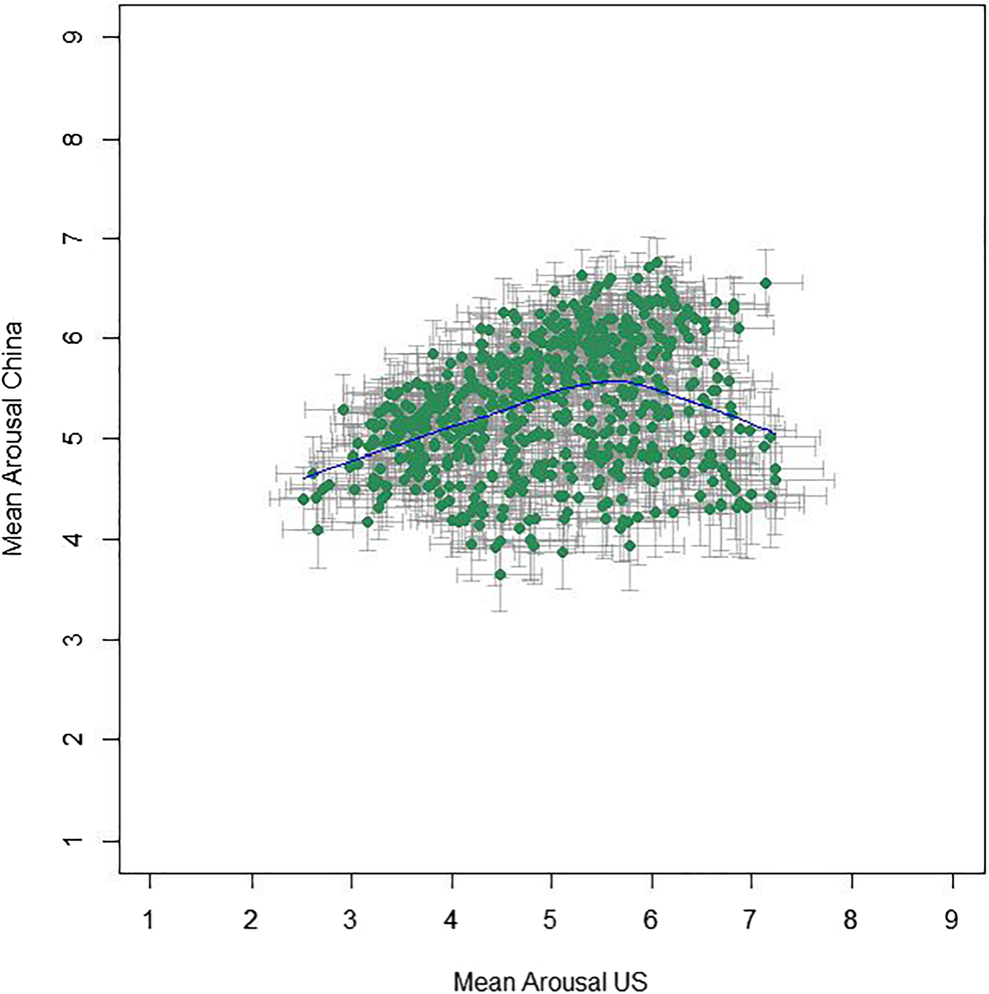
Relationship between US and Chinese mean arousal ratings (95% CI error bars) per image, with the LOWESS line in *blue*

**Fig. 8:**
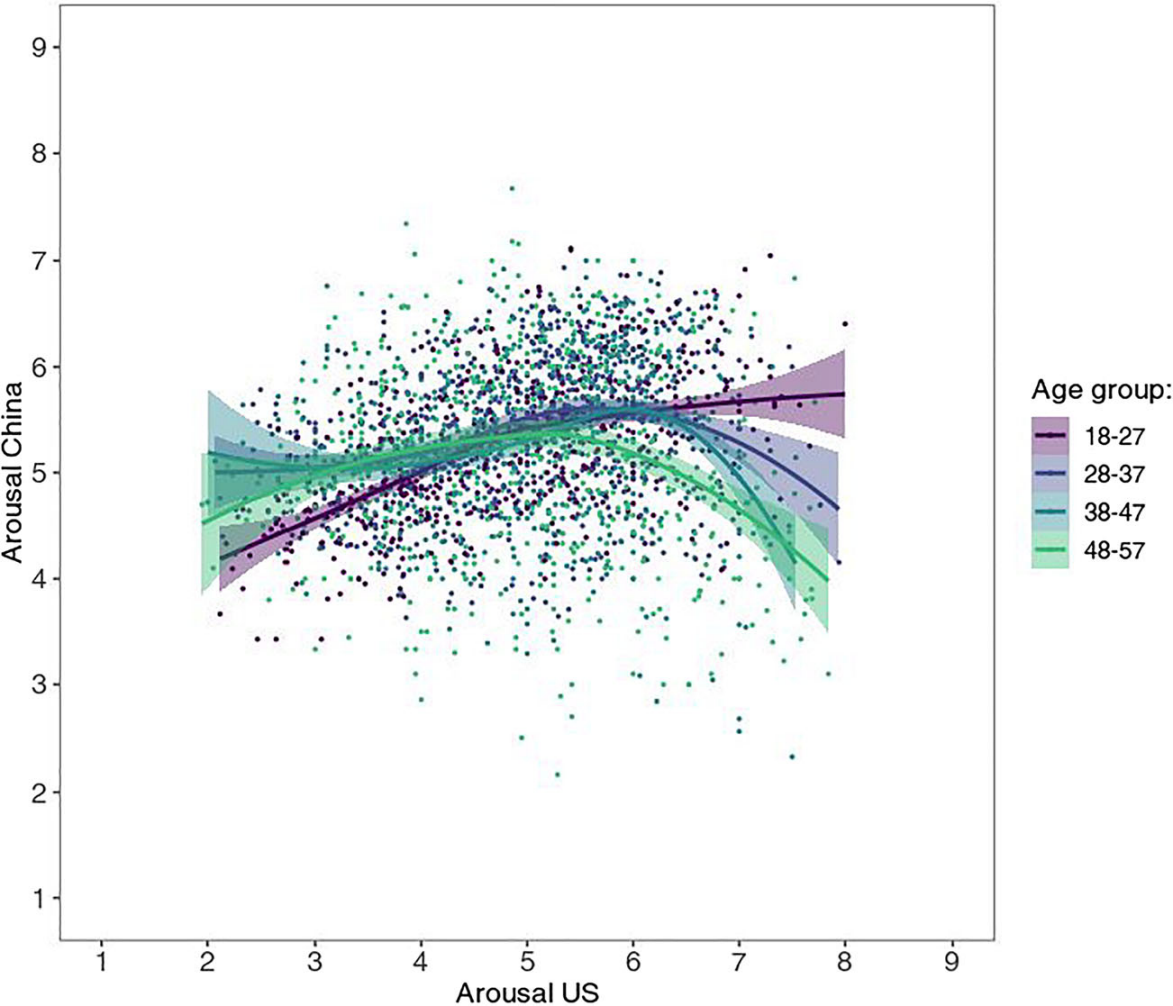
Relationship between US and Chinese mean arousal ratings per image by age group, with corresponding LOWESS lines

The mean ratings for arousal showed only a weak, albeit significant, correlation, *r*(581) = .282, 95% CI [.206, .355], *p* < .001, *BF*_10_ = 2.30 × 10^9^ (see Fig. 7). The lower correlation in the arousal (as compared to the valence) dimension relates to the fact that, as mentioned above, the data from US raters showed a U-shaped valence-arousal-relationship, but the Chinese average affective ratings displayed a linear relationship. However, just as the ratings by younger age groups showed more visually similar valence-arousal patterns (see Fig. 4), they also demonstrated substantially higher correlation with US arousal ratings, as most clearly reflected in the youngest age group (see Fig. 8; *r* = .53 as opposed to .22, .17, and – .07 for the other three age groups, see Table 2; *p* < .001 for all *z*-test comparisons; Diedenhofen & Musch, 2015). This pattern of age group influence is similar for both males and females, though the correlation was generally higher for males (*r* = .39 vs .20; Table 2; see also all figures via https://osf.io/anej6/ with male and female ratings in separate panels).

A paired *t* test showed that arousal ratings by Americans were lower than those by Chinese, again a statistically significant yet very small difference, − 0.31, 95% CI [− 0.40, − 0.23] (mean rating US: 5.00 ± 1.02, China: 5.31 ± 0.64), *t*(582) = − 7.30, *p* < .001, *d* = − 0.30, 95% CI [− 0.39, − 0.22], *BF*_10_ = 4.95 × 10^9^. This relation is similar for all age groups and both sexes (Table 2).

All in all, we may conclude that the consensus for valence ratings was generally higher than for arousal ratings, both within a sample of a given culture (see correlation tests for interrater reliability, gender, and age) and between the two cultures tested here.

## Example experiment: Color-valence congruence effects in the BASS

We also included an example experiment, in which we tested the suitability of the BASS images for online research. Here, we manipulated color to demonstrate one major advantage of the BASS images: their easy rendition in a different color. Content-wise, it has been repeatedly shown that the stimulus valence of words interacts with perceptual features like color and brightness: Positive stimuli are associated with green (rather than red) and with white (rather than black; Kawai et al., 2020; Kuhbandner & Pekrun, 2013; Lakens et al., 2012; Meier et al., 2004, 2015; Moller et al., 2009). As a validation of the suitability of the BASS for online research, in a web-based fast decision task, we tested if the BASS silhouettes can induce similar effects. Details about the experiment can be found in the online supplementary material. Below we provide only a brief summary.

### Method

We selected a subset of 60 positive and 60 negative silhouettes from the BASS (a list of filenames is provided in Table 4 in Appendix C). These 120 images were presented to 90 students (age = 23.0 ± 3.5; 27 male) from the University of Vienna in two experimental blocks: a “color” block, containing each silhouette once in red and once in green color; and a “brightness” block, containing each silhouette once in black and once in white (with silhouette background always in gray, see Appendix C, Fig. 15, for examples). The task was to categorize the valence of each silhouette via key press as either positive or negative. For each block, we ran a repeated-measures analysis of variance (ANOVA) on the correct mean response times (RTs) and another on the mean error rates (ERs).

### Results

According to expectations, the two (Color: red vs. green; within) × two (Valence: positive vs. negative; within) ANOVA in the color block showed a significant interaction between color and valence for RTs, *F*(1, 89) = 91.46, *p* < .001, ${\upeta}_{\mathrm{p}}^2$ = .507, 90% CI [.383, .595], ${\upeta}_{\mathrm{G}}^2$ = .022, *BF*_10_ = 3.10 × 10^9^, and also for ERs, *F*(1, 89) = 83.67, *p* < .001, ${\upeta}_{\mathrm{p}}^2$ = .485, 90% CI [.359, .576], ${\upeta}_{\mathrm{G}}^2$ = .106, *BF*_10_ = 2.30 × 10^15^. Positive silhouettes were categorized faster and more accurately when presented in green rather than red, and, inversely, negative silhouettes were categorized faster and more accurately when presented in red rather than green. Also, in the brightness block, the silhouettes elicited the expected brightness–valence interactions in both RTs, *F*(1, 89) = 18.68, *p* < .001, ${\upeta}_{\mathrm{p}}^2$ = .173, 90% CI [.068, .286], ${\upeta}_{\mathrm{G}}^2$ = .005, *BF*_10_ = 65.74, and ERs, *F*(1, 89) = 8.76, *p* = .004, ${\upeta}_{\mathrm{p}}^2$ = .090, 90% CI [.017, .191], ${\upeta}_{\mathrm{G}}^2$ = .011, *BF*_10_ = 7.55. Positive silhouettes were categorized faster and more accurately when presented in white rather than black, and inversely, negative silhouettes were categorized faster and more accurately when presented in black rather than white.

**Fig. 9:**
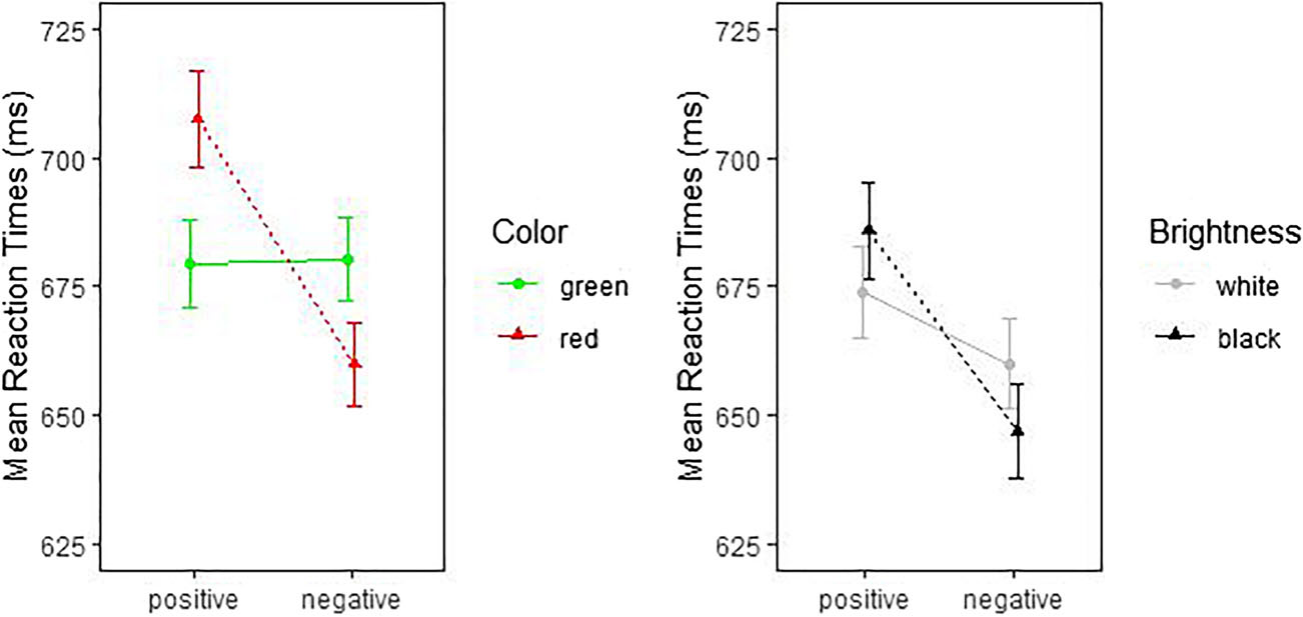
Mean reaction times for positive and negative silhouettes in the color block (*left*) and the brightness block (*right*). Error bars indicate SEMs

**Fig. 10:**
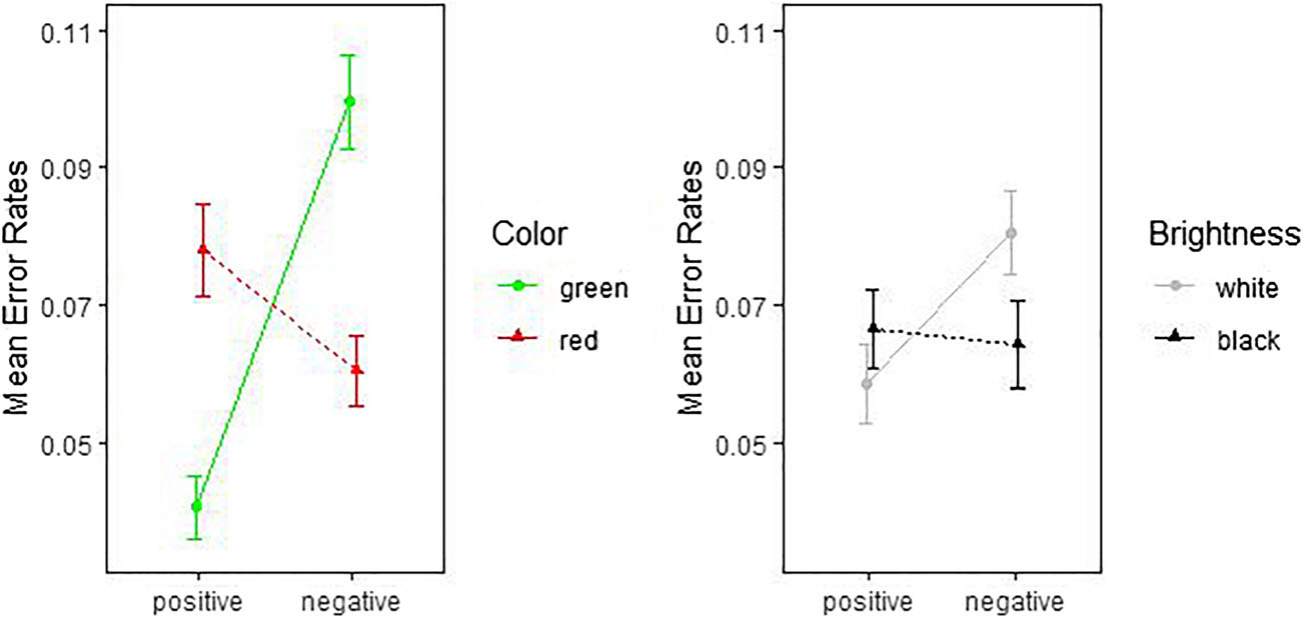
Mean error rates for positive and negative silhouettes in the color block (*left*) and the brightness block (*right*). *Error bars* indicate the SEMs

**Fig. 11:**
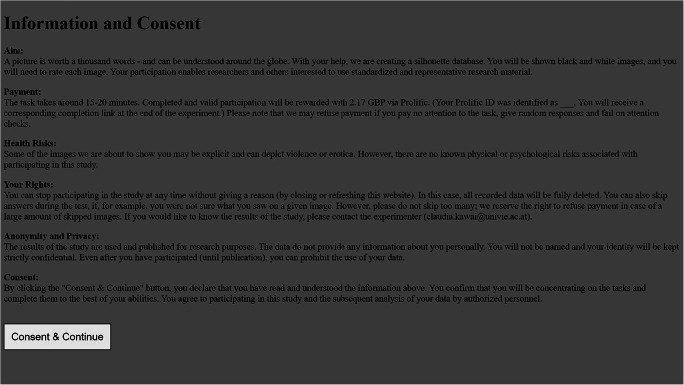
Screenshot of the experiment’s welcome page

**Fig. 12:**
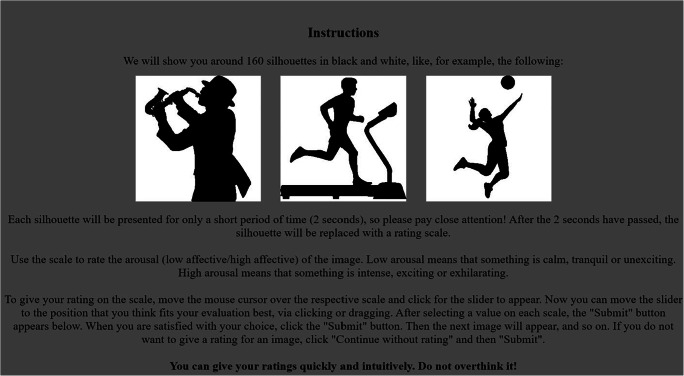
Screenshot of the instruction page after consenting (example from the arousal substudy)

**Fig. 13:**
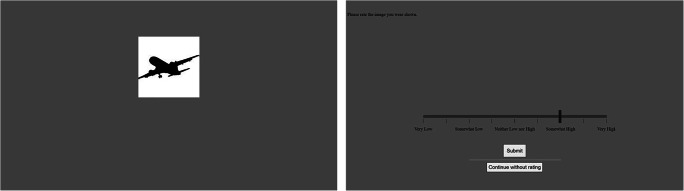
Screenshot of the presentation of the silhouette stimulus (start of the trial), followed by the appearance of the rating scale (example from the arousal substudy)

**Fig. 14:**
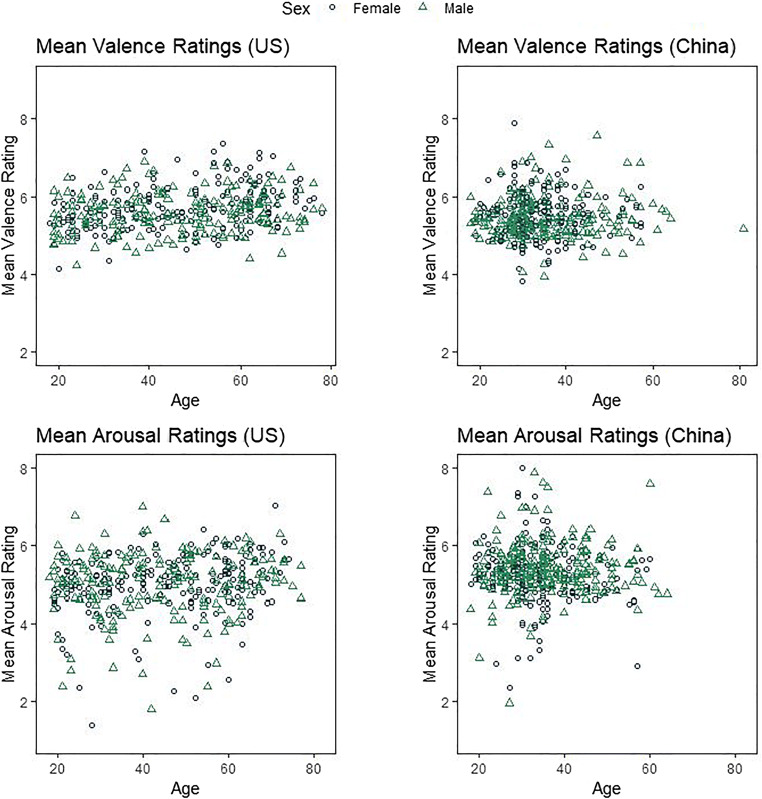
Mean valence and mean arousal ratings per participant according to their country, sex, and age group

**Fig. 15:**
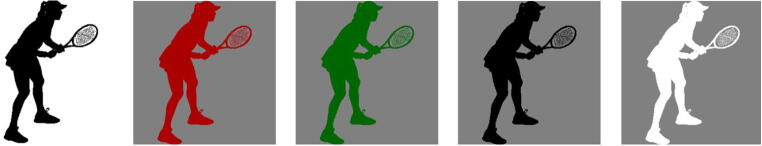
Example for color-manipulated silhouettes (from *left to right*): original, red-gray, green-gray, black-gray, and white-gray (as used in the example experiment)

Means and *SD*s for RTs and ERs are illustrated in Figs. 9 and 10, and the means can be found in Table 5.

## Discussion

For the current study, we created an affective silhouette database with the specific goal of providing an easy and openly accessible lightweight stimulus pool capable of reliable elicitation of affective representations on a wide range of valence and arousal levels that was validated by extensive samples of two of the biggest culture groups studied in psychology. In the US sample, we found that the images cover a very wide range of affective representations from very negative to very positive (valence), and from very calm to very intense (arousal), indicating various potential applications (see Introduction). The Chinese sample was remarkably similar in valence ratings, covering a wide range with an extremely high correlation with the US sample.

However, the Chinese *arousal* ratings were only weakly correlated, and comparatively limited in range. This relates to the fact that data from the US participants demonstrated a U-shaped relationship between valence and arousal, while data from the Chinese participants, instead, displayed a more linear valence–arousal relationship (strong positive correlation between valence and arousal ratings) on average.

Studies that look at affective ratings for emotional *words* in Chinese find a U-shaped valence-arousal relation similar to the one apparent in the US ratings (Ho et al., 2015; Liu et al., 2018; Yao et al., 2017). It is noteworthy, that participants in the affective word studies were high school or university students. Our Chinese sample was more diverse: The age-group-wise analysis shows that younger Chinese display a more quadratic relationship which becomes more linear with increasing age. This provides a plausible reconciliation of these previous findings with our results in that it is only the relatively older Chinese generations whose perception substantially differs from those in Western populations, while the younger generations perceive images more in accordance with the Western population. We can only speculate about the reasons, but the more similar ratings of young US and Chinese participants could reflect a larger shared sphere of visual experiences, for instance, through digital social media (e.g., TikTok).

However, two studies that compared affective ratings for emotional *photographs* (a subset of the IAPS) between Chinese and Western samples interestingly seem to indicate the opposite phenomenon: Gong and Wang (2016) found a high correlation of valence and arousal ratings between Chinese and German older adults^12^, and Huang et al. (2015) argued that Chinese young adults display a different rating behavior from US young adults for IAPS pictures.

Generally, more empirical data on cultural differences in emotion representation or judgments is necessary to establish how and when Chinese rating behavior differs from that of a Western culture. One possibility might be, for instance, that the lower arousal for negative affective silhouettes, which explains most of the difference in our BASS ratings (and the lack of U-shape among Chinese participants, especially with increasing age), might be a result of conflict avoidance or emotion control as a more generally accepted strategy among Chinese than Westerners (Gong & Wang, 2016; Tjosvold & Sun, 2002).

We also demonstrated how the BASS images could be used in online experiments. In our example experiment, we showed the typical color-congruence effects of stimulus color on speed and accuracy of judgments about image valences: Positive images were rated faster and more accurately if presented in green than in red, and if presented in white than in black, while the opposite held true of negative images.^13^ This result replicated prior research and already proves at least one particular use of the BASS images in scientific research.

All in all, the quality of our samples’ data (as measured by the respective interrater correlation) is outstanding and, where comparable, its general characteristics are in line with effects observed in notable affective databases like IAPS, OASIS, ANEW and BAWL-R (Võ et al., 2009), to name a few.

The BASS, however, aims only to complement, and not to replace, realistic picture databases, for research for which realistic images do not provide ideal stimuli for a given experiment for any of the reasons detailed in the Introduction (e.g., complications of color manipulation, file size, etc.).

While the simplicity of silhouettes can be advantageous for some research designs, their innate lack of context information may simultaneously lead to more (or less) ambiguity in their interpretations between participants than is the case for pictures that are rich in detail. Heterogeneous cultural backgrounds between participant groups could add to differences in content evaluation if contexts interact with objects in photorealistic image sets, but not or at least less so with silhouettes where context is sparse. However, while the direct interpretation of some of the silhouettes may, thus, be more or less ambiguous than that of a photographic image of the corresponding content (e.g., some participants may perceive a coyote’s silhouette simply as a dog when presented without a fitting habitat as context), in the current study, the affective ratings of the silhouettes generally demonstrated very high consistencies (very high inter-rater correlation, low *SD*s, and, in case of valence, very high intercultural correlation), which attest to the silhouettes’ emotional unambiguousness. Furthermore, several numeric measures in our BASS database, such as the rating differences between US and China per image, the corresponding *BF*s or residuals, or simply the *SD*s of the ratings in either sample, allow researchers to choose images of emotional clarity or culture-neutrality suiting their needs best.

Nonetheless, and although the rating data of our BASS images correspond well with the data obtained for photographic databases, we cannot rule out that the silhouettes evoke smaller emotional responses than contextually richer photographic pictures when using other measures (e.g., skin conductance, heart rate, facial muscles activities). Do photorealistic pictures elicit stronger emotion-related physiological responses than silhouettes, and if so, what are the driving forces in such a case (object detail, color, depth and perspective, etc.)? We hope that future research, facilitated by the BASS, will help to address these questions.

## Conclusions

The BASS offers a novel database of visual silhouettes for psychological research optimally suited for the study of image-elicited affective representations in online experiments and validated by normative ratings by representative Western and Asian samples.

## Appendix A

**Table 1:** Demographics of the included participants from the US and Chinese ratings with corresponding means and *SD* for valence and arousal

	US	Mean Valence Rating (*SD*)	Mean Arousal Rating (*SD*)	China	Mean Valence Rating (*SD*)	Mean Arousal Rating (*SD*)
**Sex**						
Female	402	5.70 (2.39)	5.00 (2.25)	419	5.49 (1.97)	5.25 (1.84)
Male	375	5.58 (2.17)	4.99 (2.28)	450	5.45 (1.94)	5.38 (1.82)
**Sum**	**777**			**869**		
**Age**						
18–27	138	5.41 (2.23)	4.85 (2.24)	186	5.42 (1.99)	5.23 (1.85)
28–37	147	5.55 (2.13)	4.90 (2.33)	419	5.48 (1.97)	5.38 (1.9)
38–47	132	5.66 (2.36)	5.04 (2.21)	176	5.47 (1.94)	5.32 (1.68)
48–57	134	5.70 (2.26)	4.96 (2.39)	74	5.50 (1.85)	5.20 (1.75)
58+	226	5.78 (2.37)	5.17 (2.16)	14	5.47 (1.78)	5.45 (1.74)
**Ethnicity**						
Asian	58					
Black	106					
Mixed	35					
Other	30					
White	548					

**Table 2:** US and Chinese valence and arousal rating correlation per sex and age group

	Valence correlation [95% CI]	Arousal correlation [95% CI]
**Sex**		
Female	.913 [.899, .926]	.198 [.119, .275]
Male	.942 [.933, .951]	.387 [.316, .454]
**Age**		
18–27	.910 [.895, .923]	.530 [.469, .586]
28–37	.921 [.908, .933]	.219 [.140, .295]
38–47	.899 [.882, .913]	.170 [.090, .248]
48–57	.856 [.833, .876]	– .069 [– .150, .012]
58+ *	.689 [.644, .730]	.251 [.173, .326]

* This oldest age group includes only 14 Chinese participants (cf. Table 1), hence the related correlations are not reliable.

## Appendix B

**Table 3:** Bicolor Affective Silhouettes and Shapes (BASS) datatable manual

Column	Explanation
file_name	The image file name.
val_mean_us	The mean of all valence ratings.
aro_mean_us	The mean of all arousal ratings.
val_sd_us	The *SD* of all valence ratings.
aro_sd_us	The *SD* of all arousal ratings.
val_n_us	The number of valence ratings.
aro_n_us	The number of arousal ratings.
val_cilo_us	Lower limit of 95% CI for the mean of all valence ratings.
val_ciup_us	Upper limit of 95% CI for the mean of all valence ratings.
aro_cilo_us	Lower limit of 95% CI for the mean of all arousal ratings.
aro_ciup_us	Upper limit of 95% CI for the mean of all arousal ratings.
val_diff	The difference between the means of US and Chinese valence ratings. (val_mean_us minus val_mean_ch).
aro_diff	The difference between the means of US and Chinese arousal ratings. (aro_mean_us minus aro_mean_ch).
val_bf_10	Wilcoxon Bayes factor in support of difference between US and Chinese. valence ratings.
val_bf_01	Wilcoxon Bayes factor in support of equivalence between US and Chinese. valence ratings.
aro_bf_10	Wilcoxon Bayes factor in support of difference between US and Chinese. arousal ratings.
aro_bf_01	Wilcoxon Bayes factor in support of equivalence between US and Chinese. arousal ratings.
val_resid	Linear regression residuals for valence ratings: observed values minus fitted. values for the US-Chinese mean ratings relations for the entire sample. A larger value means larger US mean valence rating than Chinese mean valence rating, relative to what is expected when values are perfectly correlated (linear fitted values). The closer this value is to zero, the more likely it is that the given image is suitable for cross-cultural measurement of valence. (Nonetheless, this value is quite close to val_diff, which is more straightforward to interpret.)
aro_resid	Linear regression residuals for arousal ratings (same as for valence, see above).
black_px	Number of black pixels in the image.
white_px	Number of white pixels in the image.
keywords	Keywords for the given image.
source	Source of the (original) image the silhouette is based on.

*Note.* The columns listed above with “_us” suffixes refer to rating data from the US sample. The data referring to the Chinese ratings is indicated with "_ch" suffixes – here omitted for brevity. (E.g., val_mean_ch: The mean of all valence ratings from China, etc.)

## Appendix C

**Table 4:** Filenames of the positive and negative silhouettes used in the verification study

Positive silhouettes		Negative silhouettes	
trapeze4	badminton	injury	handinjury
wedding2	jump	attack2	grenade
love	highjump4	execution3	robber
singer3	highjump	abuse2	bazooka
couple6	acrobat3	abuse	pollution2
romance	couple5	hanging	gunman
volleyball	skiing2	drunkard	drunk
champion	dance2	vomit2	tick
euphoria	ferriswheel	argument4	axe3
iceskater3	concert	handcuff2	scorpion2
success2	iceskater	noose	mosquito
affection2	skateboard	argument3	gasmask2
cardio	trapeze3	argument2	handcuff
handball	tennis	dispute	knife2
kiss4	handball2	argument6	pollution
ballet	winner	hazard	mosquito2
basketball4	rockstar	hitman	knife4
propose	carefree	sorrow2	snake2
kiss3	basketball	murder2	wasp
joy	breakdance	gunman2	machete
acrobat	skiing3	beggar2	tank2
trapeze	soccer	skull2	soldier2
jump2	aerobics	scolding2	gun
happiness	highjump2	gunman3	kalashnikov
surfer2	snowboard2	knife	tank
skiing	couple7	axe	wildwest
soccer2	gymnastics3	accident2	sniper
father2	highjump3	roach2	rifle
propose3	dog3	vomit	skull
skateboard2	poledance	falling6	snake

**Table 5:** Means and *SD*s for reaction times (RTs) and error rates (ERs) per color and valence categories in the Bicolor Affective Silhouettes and Shapes (BASS) validation experiment

Color	Valence	RT *M* ± *SD*	ER *M* ± *SD*
Red	Negative	659.96 ± 200.61	0.06 ± 0.24
Red	Positive	708.00 ± 233.20	0.08 ± 0.27
Green	Negative	680.91 ± 207.14	0.10 ± 0.30
Green	Positive	679.24 ± 217.30	0.04 ± 0.20
Black	Negative	647.60 ± 202.37	0.06 ± 0.25
Black	Positive	686.14 ± 217.78	0.07 ± 0.25
White	Negative	660.16 ± 202.40	0.08 ± 0.27
White	Positive	674.30 ± 210.28	0.06 ± 0.24

## Footnotes

1 Some of the BASS images are not *silhouettes* in the strictest sense, but rather *silhouette-like shapes* because they also illustrate hollow parts or outlines (e.g., the edges and dots on dice). Nonetheless, for simplicity, in the present article, we refer to all the BASS images as “silhouettes.”

2 Several databases of shapes or objects do exist, like MultiPic (Duñabeitia et al., 2018), ALOI (Geusebroek et al., 2005), the sets by Nishimoto et al. (2012), by Brady et al. (2013), or by Snodgrass and Vanderwart (Rossion & Pourtois, 2004; Snodgrass & Vanderwart, 1980). However, these were not created (let alone verified or normed) with the intention of eliciting affect: The majority of these images depict everyday objects or animals. With a few exceptions, like dangerous versus cute animals, or weapons, it is unlikely that they would elicit strong affective responses or emotions. In the OASIS, for example, people and scene pictures were rated as more emotional than objects (Kurdi et al., 2017, p. 463), which suggests that these databases are not ideal for emotion research purposes.

3 IAPS: US undergraduate students, OASIS: more diverse but predominantly white US participants, GAPED: South/West-European psychology students, NAPS: Polish and other mostly European students.

4 An R script for easy instant conversion of any number of images to any chosen color is available via https://gasparl.github.io/BASS/.

5 The contents of most BASS images can be easily recognized even when they are strongly blurred or scaled to icon size.

6 For two US participants, we do not know their definite ages, only which age group they belong to. Mean age and *SD* are calculated without these two participants.

7 Duration ranged from 10 min to 53 hours. The latter was an extreme outlier that is no doubt due to a measurement error: For example, the participant may have opened the link to the task but only began completing it after a very long pause.

8 The demographic data for people who participated in the representative US rating was provided by Prolific.

9 This can either happen when the repetition items needed to calculate the correlation were skipped or when the same rating, for example, “5”, was given on every trial.

10 Exclusion criteria 3–4 were empirically determined by pilot testing in the pilot study, which is described in more detail in the supplementary online material.

11 The same U-shaped relationship was found in the IAPS and OASIS. Our observed valence-arousal correlation is very similar to that of the IAPS, which shows *r*(1192) = − .289, 95% CI [− .340, − .236], *p* < .001, *BF*_10_ = 2.16 × 10^21^, while the correlation in the OASIS was not significant, with *r*(898) = − .058, 95% CI [− .123, .007], *p* = .082, *BF*_01_ = 2.84.

12 Gong and Wang (2016) compared their Chinese participant sample with a mean age of 67.3 ± 4.96 years to a German participant sample with a mean age of 69.61 ± 3.58 years (Grühn & Scheibe, 2008). Thus, in this comparison, participants were older than our oldest Chinese age group (58+) which had a mean age of 62.36 ± 5.70 years and included only 14 participants.

13 The finding may seem to contradict the pilot study results showing that black-on-white and (inverted) white-on-black images are rated very similarly, demonstrating equal recognizability. However, in the pilot study, each participant was presented with only one image type (either all images black-on-white, or all white-on-black). Color-valence congruence effects can depend on dichotomous decision alternatives within the same task (cf. Kawai et al., 2020; Lakens et al., 2012), and, the present results are in line with this possibility and previous research showing that such congruence effects were only elicited in the example experiment (where each task contained contrasting color types alternating trial by trial), not during the affective rating study.
